# Acid-Producing Diet and Depressive Symptoms among Breast Cancer Survivors: A Longitudinal Study

**DOI:** 10.3390/cancers12113183

**Published:** 2020-10-29

**Authors:** Tianying Wu, Fang-Chi Hsu, John P. Pierce

**Affiliations:** 1 Division of Epidemiology and Biostatistics, School of Public Health, San Diego State University, San Diego, CA 92182-4162, USA; 2 Moores Cancer Center, School of Medicine, University of California at San Diego, San Diego, CA 92093, USA; jppierce@ucsd.edu; 3 Department of Biostatistics and Data Science, Division of Public Health Sciences, Wake Forest School of Medicine, Winston-Salem, NC 27157, USA; fhsu@wakehealth.edu

**Keywords:** dietary acid load, depression, breast cancer survivors

## Abstract

**Simple Summary:**

Depressive symptoms, which are highly prevalent among breast cancer survivors, can significantly influence quality of life and increase total mortality. The aim of our prospective study was to determine whether acid-producing diets have an adverse impact on depression. Our study demonstrated that a higher consumption of acid-producing diets was significantly associated with depressive symptoms among breast cancer survivors, especially among those who were younger than 55 and had a sedentary lifestyle.

**Abstract:**

The incidence of depression is two-to-three times higher in cancer survivors than the general population. Acid-producing diets may play important roles in the development of depression. Cancer survivors are more susceptible to acid-producing diets, yet few prospective studies have investigated the association of acid-producing diets with depression among breast cancer survivors. We leveraged a large cohort of 2975 early stage breast cancer survivors, which collected detailed dietary data via 24-h recalls. Potential renal acid load (PRAL) and net endogenous acid production (NEAP), two commonly used dietary acid load scores, were used to estimate acid-producing diets. Intakes of PRAL and NEAP were assessed at baseline and years one and four. Increased PRAL and NEAP were each independently associated with increased depression in the longitudinal analyses, after adjusting for covariates. The magnitude of the associations was stronger for PRAL than NEAP. Women with the highest quartile intakes of PRAL had 1.34 (95% CI 1.11–1.62) times the risk of depression compared to women with the lowest quartile. Furthermore, we also observed a joint impact of PRAL and younger age on depression, as well as a joint impact of PRAL and physical activity on depression. Decreasing the consumption of acid-producing diets may be a novel and practical strategy for reducing depressive symptoms among breast cancer survivors, especially those who are younger and have a sedentary lifestyle.

## 1. Introduction

The incidence rate for depression is two-to-three times higher in the cancer population compared to their healthy counterparts [1]. Depression has been shown to be strongly associated with poor quality of life [2], all-cause mortality [3], and cancer-specific mortality [4,5]. Thus, the 2016 US Preventive Services Task Force Statement recommended depression screening and follow-up in all adults and emphasized that cancer survivors face higher risks of depression [6]. Some possible risk factors have been suggested for depression in cancer survivors [7]. For instance, increased glutamate released from cancer cells can lead to excitotoxicity [8], tissue destruction due to surgery or chemotherapy can lead to inflammation [9,10], and stress caused by cancer diagnosis and treatment can disrupt the hypothalamic–pituitary–adrenal (HPA) axis [11]. Observational studies have shown that metabolic syndrome [12], social support [13,14], severe life difficulties [9], and poor diet quality [15] were also associated with depression in cancer survivors.

A large body of prospective studies has shown that dietary factors play important roles in the development of depression in the general healthy population [16,17]; however, few longitudinal studies have examined the associations of acid-producing diets with depression among cancer survivors. Among the general heathy population, adherence to a high-quality diet such as a prudent/Mediterranean diet has been associated with a lower risk of depression in a linear dose-response pattern [16,18,19]; vegetable consumptions and a low dietary inflammatory index were somewhat associated with a lower incidence of depression [16,20,21], though not in a dose-response fashion [16]. Although adherence to low-quality diets and food groups did not appear to be associated with higher depression incidence in the general healthy population [16], cancer survivors may be more susceptible to an unhealthy diet, as they have a higher risk of depression than their general healthy population counterparts [1]. However, studies examining unhealthy dietary factors associated with depression in cancer survivors, especially breast cancer survivors, are limited. Breast cancer is the most common cancer in American women [22]. A cross-sectional study demonstrated that a low-quality diet was associated with depression in breast cancer survivors [15]; however, longitudinal studies examining the impact of dietary patterns with depression among breast cancer survivors are limited.

Cancer patients are more susceptible to acid-producing diets than healthy populations without cancer, as cancer survivors have a reduced capacity to adjust their acid-base balance [23]. We demonstrated that, among breast cancer survivors, acid-producing diets were associated with increased inflammation and hyperglycemia [24], both of which are risk factors for depression [10,25]. A diet high in alkaline foods, such as fruits and vegetables, but low in acid-producing foods, such as meat and cheese, can lower endogenous acids production, whereas a Westernized diet, i.e., high in proteins, meat, cheese, refined carbohydrates, and soft drinks and low in fruits and vegetables, can induce endogenous acid production [26,27]. Until now, few studies have examined the association between an acid-producing diet and depressive symptoms among breast cancer survivors or any types of cancer survivors.

The central objective of this study is to determine whether acid-producing diets are associated with increased depression in a longitudinal study. The Women’s Healthy Eating and Living (WHEL) Study collected detailed dietary information and assessed depressive symptoms prospectively and, thus, provided an opportunity to investigate whether acid-producing diets predicted depression in a large cohort of breast cancer survivors who were enrolled up to four years after diagnosis.

## 2. Materials and Methods

### 2.1. Study Design

The detailed inclusion and exclusion criteria for the WHEL study were described previously [28]. Briefly, breast cancer survivors enrolled in the WHEL study were recruited from multiple clinical centers located in California, Arizona, Texas, and Oregon. Between 1995 and 2000, 3088 early stage (stage I, II, or IIIA) breast cancer survivors within 4 years of diagnosis were recruited for a clinical trial. The clinical trial in the WHEL study aimed to test whether a diet high in fruits, vegetables, and fiber and low in total fat would improve breast cancer prognosis [28]. The average follow-up time of this trial was 7.3 years. Since the original trial did not significantly change breast cancer prognosis by the end of the follow-up [29], the current study treated and analyzed the WHEL data as a cohort. For the current study, we included 2975 participants who provided data on both diets and depressive symptoms.

This study was approved by the Institutional Review Board (IRB) at the University of California at San Diego and other participating centers, including the University of California at Davis, Stanford University, University of California at San Francisco, the University of Arizona, MD Anderson Cancer Center, Kaiser Permanente Norther California, and the Center for Health Research, Portland, Oregon. Since we used the deidentified data provided by the principal investigator of the WHEL study, we received the exempt IRB by the San Diego State University (protocol number: Temp-1286).

### 2.2. Dietary Assessment

The prescheduled 24-h recalls were collected 4 times by telephone on random days over a 3-week period, including two on the weekends and two during weekdays. The four 24-h recalls were conducted at baseline, year 1, and year 4. The multi-pass Minnesota Nutrition Data System software was used for calculating the amount of each nutrient and foods, such as vegetables (NDSR, 1994–2006, University of Minnesota, Minneapolis, MN, USA).

To assess the consumption of acid-producing diets, we use two commonly used dietary acid load scores: the net endogenous acid production (NEAP) score and the potential renal acid load (PRAL) score. NEAP is a simple algorithm developed by Frassetto et al. that includes only proteins and potassium [30], whereas the PRAL algorithm developed by Remer and Manz takes into account other pH-altering nutrients, including calcium, magnesium, and phosphate [31]. We can use the scores of NEAP and PRAL to evaluate an overall acid-producing potential for foods consumed by each participant. For instance, a lower NEAP score and a negative PRAL score mean that a diet has alkaline-producing potential, whereas a higher NEAP score and a positive PRAL score indicate that a diet has an acid-producing potential. The formulas for calculating NEAP and PRAL scores are displayed below [32].

NEAP (mEq/d) = (54.5 × protein [g/d]/potassium [mEq/d]) − 10.2(1)

PRAL (mEq/d) = (0.49 × protein [g/d]) + (0.037 × phosphorus [mg/d]) − (0.021 × potassium [mg/d]) − (0.026 × magnesium [mg/d]) − (0.013 × calcium [mg/d])(2)

### 2.3. Assessment of Depression Symptoms

The Center for Epidemiologic Studies Depression Scale (CES-D) was one of most popular measures of depressive symptoms [33,34]. The CES-D instrument was administered in the WHEL study. The following items were used to evaluate the frequency of depression symptoms among participants in the past week: (1) you felt depressed, (2) your sleep was restless, (3) you enjoyed life, (4) you had crying spells, (5) you felt sad, and (6) you felt that people disliked you. Participants were scored 0 if they answered “none of the time”, 1 for “some of the time”, 2 for “a moderate amount of time”, and 3 for “most of the time”. Item 3 was reversely scored. The depression score was calculated by summing up all these item scores. The cut-off for classifying depression was ≥5, which is a standard threshold value [33,34,35,36].

### 2.4. Other Assessments

Demographic characteristics such as age, body mass index, and smoking status were collected using self-reported questionnaires; health conditions such as diabetes and cardiovascular diseases were also collected via self-reported questionnaires. Data related to cancer were extracted from medical records, which included initial cancer diagnosis and treatment, hormone receptor status, tumor stage, size, and use of chemotherapy and tamoxifen. We used a validated questionnaire to assess the physical activity [37], which was expressed as metabolic equivalent tasks (METs), as previous studies did [38].

### 2.5. Statistical Analyses

All analyses were conducted using SAS version 9.2 (SAS Institute, Cary, NC, USA). We conducted descriptive statistics to compare the differences in baseline characteristics across the depression status (CES-D ≥ 5 vs. CES-D < 5) and across the baseline dietary acid load (PRAL). We used a *t*-test and analysis of variance (ANOVA) for normally distributed continuous variables and Wilcoxon rank-sum test and Kruskal-Wallis test for non-normally distributed continuous variables. Chi-Square was used for categorical variables.

The dependent variable, depression, was treated as a dichotomized variable (CES-D ≥ 5 vs. CES-D < 5). To account for the correlations among repeated assessments within an individual, the marginal models incorporating generalized estimating equations (GEEs) were used (Proc GENMOD in SAS) to determine the longitudinal associations between dietary acid load scores (PRAL and NEAP) and depression. The exposures of interest were time-varying dietary acid load scores, which were measured at years 0, 1, and 4. We used the average levels of dietary acid load scores at years 0, 1, and 4 to set up the cut-off point for each quartile of PRAL and NEAP. Since the WHEL study was originally a dietary intervention study, PRAL and NEAP changed significantly at years 1 and 4; therefore, using baseline intakes to classify PRAL may not represent the average intakes of dietary acid load during the follow-up. Covariates in the regression models were determined based on a priori assumption; these covariates included age at diagnosis, intervention group, menopausal status, total calorie intake, smoking and pack-year status, physical activity, body mass index (BMI), living alone status, estrogen and progesterone receptor status, use of tamoxifen, chemotherapy, tumor stage, and medical comorbidities. Some of these covariates were not significantly associated with depression and did not materially change the estimates of the dietary acid load; thus, we removed them. We also included other foods or nutrients that are potentially associated with depression, such as processed and unprocessed red meats, total vegetables, and vitamin B6 in the model; as unprocessed and processed red meat were not significantly associated with depression and did not change the estimates of the exposure, they were removed from the final model. Our final model was multivariable model 2 (see Table 3), which included visit, age at diagnosis, menopausal status, total calorie intake, intakes of vitamin B6, physical activity, living alone status, BMI, and medical comorbidities at baseline. Although the total vegetable intake was found to be inversely associated with depression and was adjusted in the multivariable model 3 (see Table 3), it was not included in the final model. The reasons for not including the total vegetable intake were as follows: PRAL is a summary score of acid- and alkaline-producing foods, and vegetables are the main alkaline-producing foods; the correlation between total vegetable intake and PRAL was moderately inverse (r = −0.5; *p* < 0.0001). The aim of adjusting for total vegetable intakes in multivariable model 3 was to demonstrate that the association between PRAL and depression was independent of the vegetable intake; in other words, it was not due to the total vegetable intake.

Among the covariates in the multivariable models, time-varying covariates included the BMI, physical activity, living alone status, total calorie intake, and intakes of vitamin B12. We used baseline data for other covariates. The *p*-value for the linear trend test for each dietary acid load score was presented. To be conservative, as we presented two dietary acid load scores, we used Bonferroni correct to set up the significance at 0.025 (0.05/2)

Using the GEE model, we also examined the joint impacts of dietary acid load with age and activity. For instance, the age of diagnosis was divided into three categories (<47, 47–55, and 55+). Two groups of PRAL were created using the median as the cut-off point. Six joint groups of PRAL with age categories were created. In multivariate model 2, we examined the associations of the 6 joint categories with depression. The odds ratio (95% CI) was presented.

## 3. Results

### 3.1. Baseline Characteristics by Disease Outcomes in the Whole Cohort

As shown in Table 1, 614 women had depression (CES-D score ≥ 5) at baseline. Women who had a diagnosis at an older age, had education above the college level, and were engaged in more physical activities, were in the menopausal stage, and used tamoxifen were less likely to have depression symptoms (*p*-values were ≤ 0.05 for all these comparisons). Women who had the higher PRAL and NEAP scores, were overweight or obese, and abstained from alcohol were more likely to have depression (*p*-values ≤ 0.05 for all these comparisons).

### 3.2. Baseline Characteristics by Quartile of PRAL

Table 2 displays the associations of baseline characteristics with PRAL. We found that, compared to women with a PRAL score, women with a higher PRAL score were more likely to be younger, overweight and obese; had a higher range of depression scores; and higher intakes of vitamin B12 and lower intakes of vegetables; they were less likely to be postmenopausal women and tamoxifen users and have positive ER or PR status. PRAL was negatively associated with levels of physical activity and higher education. *p*-values were <0.05 for these comparisons. 

### 3.3. Longitudinal Associations between Dietary Acid Load and Depression (CES-D Score)

There were 614, 434, and 427 depression cases at baseline, year one, and year four, respectively. Depression, which was expressed by a CES-D score, was analyzed as a binary variable. As shown in Table 3, the positive associations of dietary acid load with depression were statistically significant for PRAL and marginally significant for NEAP when we compared differences between the extreme quartiles of PRAL. *P*-values for the trends were significant for both PRAL and NEAP. The magnitude of the associations was attenuated in multivariable-adjusted models compared to age-adjusted models and were stronger for PRAL than NEAP. In the final model (multivariable model 2), the additional adjustment of vitamin B12 intake slightly improved the estimates for PRAL and NEAP, whereas the additional adjustment of both vegetable and vitamin B12 intakes in multivariable model 3 slightly attenuated the estimates for PRAL and NEAP.

### 3.4. Joint Impacts of Dietary Acid Load and Age on Depression, and Joint Impacts of Dietary Acid Load and Physical Activity on Depression

**Figure 1:**
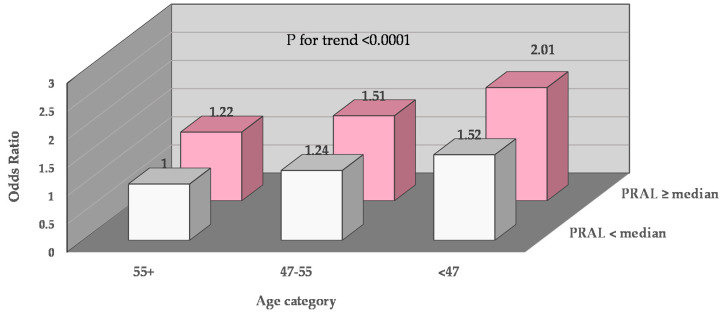
Joint associations of PRAL and age with depression. Covariates in the multivariable model included the body mass index, physical activity, living alone status, menopausal status at baseline, number of comorbidities at baseline, total calorie intake, and intakes of vitamin B12. Abbreviations: PRAL: potential renal acid load.

In multivariate model 2 used in Table 3, we examined the joint associations of PRAL and age with depression (CES-D ≥ 5 vs. CES-D < 5). As shown in Figure 1, women with age at diagnosis <47 and PRAL intakes higher than the median had two times the risk of depression (odds ratio = 2.01; 95% CI 1.57–2.57) when compared to women with ages ≥55 and PRAL intakes less than the median (*p*-for-trend < 0.0001). Similarly, we examined the joint association of PRAL and physical activity in multivariable model 2. As shown in Figure S1, women whose activity level was lower than the median and PRAL intakes were higher than the median and experienced depression two point four times more often (odds ratio = 2.41; 95% CI, 1.85–2.91) than women who had an activity level higher than the median and PRAL intakes lower than the median.

## 4. Discussion

Our study is the first longitudinal study among breast cancer survivors to demonstrate that acid-producing diets were associated with depression; the associations were stronger in women who had breast cancer diagnosed before age 55. In addition, we compared women who had two features—namely, a dietary acid load score higher than the median and a diagnosis before age 47—to women without these the two features (i.e., a dietary acid load score lower than the median and a diagnosis after age 55); the former had two times the risk of depression (odds ratio = 2.01; 95% CI, 1.57–2.57) than the latter group. Moreover, women whose PRAL intakes were higher than the median and activity level was lower than the median had more than two times the risk of depression (odds ratio = 2.41; 95% CI, 1.85–2.91) than women who had PRAL intakes lower than the median and an activity level higher than the median.

To our knowledge, no other prospective studies have examined the associations of dietary acid load with depression among breast cancer survivors. Our study is the first study to demonstrate a strong association between dietary acid load and depression among breast cancer survivors; we also demonstrated that the magnitudes of the associations were stronger for PRAL than NEAP. A few studies have examined the associations of dietary acid load with depression or mental health outcomes among noncancer populations [39,40,41]. One cross-sectional study among diabetic women in Iran demonstrated that dietary acid load was associated with depression; the magnitude of the association was stronger for PRAL than NEAP [39]. Although this study was among diabetic women and was cross-sectional, the trends of their findings were in-line with ours. We found similar trends: When comparing the highest to the lowest quartile, PRAL was associated with a 17% higher risk of depression than NEAP. Another study conducted among children in Germany analyzed both the cross-sectional and prospective associations between PRAL and depression [41]. This study found that PRAL was associated with emotional problems both cross-sectionally and prospectively [41]. Our study, as well as the previous two studies [39,41], both suggest that the PRAL score is a better predictor for depression than NEAP. On the other hand, one cross-sectional study among noncancer adults (men and women) in Iran demonstrated a strong association of NEAP with depression (OR = 1.56; 95% CI 1.27–1.95, comparing the highest to the lowest category of NEAP) [40]. However, this study did not adjust for the living alone status or intakes of vitamin B12, which were all significantly associated with depression in our study and some other studies [21,42,43] and can potentially confound the results. Nevertheless, none of these studies were conducted among cancer survivors, who may have different metabolisms as compared to noncancer survivors. Thus, more longitudinal studies among breast cancer survivors are needed to confirm our results.

Several biological mechanisms may help explain why acid-producing diets are associated with depression in cancer survivors. Cancer or the cancer treatment itself can damage human bodily systems (electrolytes, respiratory system, kidneys, or bones) that are needed to adjust the acid-base balance [23]. Hence, cancer survivors are more susceptible to acid-producing diets than the general population, as they have a lower capacity to adjust the acid-base balance than the general population. Further, acidity is involved in the development of neuropathy [44]. A higher dietary acid load or stress caused by cancer diagnosis or treatment can increase levels of glucocorticoid [45,46,47], which have been found to lead to brain atrophy and the dysfunction of glutamatergic neurotransmission [47,48]. Neuropathy, brain atrophy, and the dysfunction of glutamatergic neurotransmission were found to be associated with the development of depression [47,49,50]. Moreover, we demonstrated that a dietary acid load was associated with inflammation and hyperglycemia [24]; inflammation and hyperglycemia have been proposed to be some of the etiologic factors for depression [10,25,51,52].

The potential synergy impact of PRAL and age on depression, as well as the synergy impact of PRAL and physical activity on depression, are in-line with previous observational and intervention studies and, also, support the biological mechanisms described in the previously noted studies. Prior observational studies reported that, among breast cancer survivors, younger women had a higher risk of depression than older women after breast cancer diagnosis [53,54]. Physical activity has been found to modestly reduce depression among cancer survivors in intervention studies [55,56]. Several conjectures may help explain the potential biological mechanisms. Younger women tend to be diagnosed at later stages [57] and have higher stress levels than older women due to the demands of childcare, employment, and abrupt menopause [58]. Although vigorous activity may temporarily increase endogenous acid production during the exercise period, long-term activity can improve lung function [59], which can help increased the capacity to excrete excess acid [60]. Our results supported that higher PRAL and a sedentary lifestyle have a joint impact on increasing depression. As previously explained, cancer itself, stress, and acidosis all promote the development of depression.

This study has several strengths. Our study demonstrated, for the first time, a significant and positive association between dietary acid load and depression among breast cancer survivors in a longitudinal study. The WHEL study collected four 24-h recalls during each visit (baseline, year one, and year four), which has rarely been done in other cohorts. For example, the large European Prospective Investigation into Cancer and Nutrition (EPIC), a large multicenter cohort study, only collected one-time 24-h recalls among selected participants. This unique advantage reduced within-person variability in the measure of dietary acid load scores and enabled us to assess this exposure more accurately than other cohorts. The large sample size and wide range of dietary acid load scores due to the increased consumption of fruits and vegetables in the treatment arm provided us with sufficient power to adjust multiple covariates. However, this study has several limitations. Women in this study were primarily White, and self-selected participants enrolled in a clinical trial; thus, our results may not be generalizable to women from other ethnic groups or a wide range of women with different characteristics. Our findings may not be generalizable to women who have been recently diagnosed with breast cancer, as the WHEL study enrolled women up to four years after diagnosis. Furthermore, potential residual confounding by physical activity and obesity cannot be ruled out. Physical activity has been found to modestly reduce depression among cancer survivors in intervention studies [55] and the current study; although weight loss trials have not indicated that weight reduction has a significant impact on depression among breast cancer survivors [61], we did observe a positive association between BMI and depression in the current study. To further reduce the residual confounding by activity and BMI, we examined the associations between PRAL and depression in different strata across BMI and the activity status. Our results further confirmed that the association between PRAL and depression was less likely confounded by obesity, but there may be an effect modification by activity. Our results presented in Figure S1 further confirmed effect modification by activity, as we found a joint impact of PRAL and sedentary activity on depression.

## 5. Conclusions

Our study demonstrated that the dietary acid load, particularly the PRAL score, was significantly associated with depression; this association was independent of some acid- or alkaline-producing foods, such as red meat and vegetables, and was independent of one of the depression predictors—namely, vitamin B12 [42]. Depression is common in cancer survivors and can significantly reduce their quality of life and increase all-cause mortality [2,3]. Precision care should focus on not only depression screening but, also, depression prevention. Diet is an important modifiable risk factor for depression but remains largely understudied among breast cancer survivors. Therefore, identifying the dietary acid load score as a risk factor for depression among breast cancer survivors will potentially have important implications in modifying the current dietary guidelines for cancer survivors. For instance, the American Cancer Society’s (ACS) current dietary guidelines for breast cancer survivors do not include the dietary acid load [61]. Furthermore, our study provides important messages to breast cancer survivors who are younger and those who have a sedentary lifestyle, as these women have a higher risk of developing depressive symptoms than other breast cancer survivors. Randomized trials could further confirm whether alkaline-producing diets can reduce depressive symptoms among breast cancer survivors.

## Figures and Tables

**Table 1:** Baseline characteristics of breast cancer survivors by depression symptoms. CES-D scores ≥5 indicate depressive symptoms.

	CES-D Score < 5	CES-Score ≥ 5	
	*n* = 2361	*n* = 614	*p*-Value
**PRAL (mEq/d) ^a^**	−4.49 (−14.21 to 4.15)	−1.55 (−10.47 to 7.23)	<0.0001
**NEAL (mEq/d)**	39.28 (32.16 to 47.82)	42.30 (34.23 to 50.57)	<0.0001
CES-D score	1 (1 to 3)	7 (5–8)	<0.001
Basic			
Age at diagnosis (years)	51.0 (45.0 to 58.0)	49.0 (44.0 to 55.0)	<0.0001
White (%)	85.4	82.4	0.06
Body mass index			
Normal weight (%)	44.4	36.0	<0.0001
Overweight and obese (%)	55.6	64.0	
Education, at or above college (%)	54.9	50.3	0.05
Postmenopausal women (%)	80.5	75.5	0.002
Smoking status			
Past smoker (%)	41.0	42.5	0.1
Never smoker (%)	54.0	51.3	
Pack-year status			
Pack-years = 0 (%)	54.5	51.5	0.1
Pack-years > 0 to 15 (%)	24.7	31.2	
Pack-years > 15 (%)	17.3	16.2	
Alcohol abstainer (%)	30.4	34.5	0.05
Physical activity (MET/week)	675 (225 to 1350)	420 (105 to 1000)	<0.0001
Intervention group (%)	49.7	49.0	0.8
Chemotherapy (%)	69.1	71.6	0.4
Radiation (%)	61.2	62.8	0.3
Hormone receptor status			
ER+/PR+ (%)	62.3	61.0	0.2
ER−/PR− (%)	21.2	24.5	
Cancer stage at diagnosis (%)			
I	38.0	41.2	0.3
II	57.2	53.4	
IIIa	4.9	5.4	
Tamoxifen use (%)	67.7	61.0	0.007

^a^ Continuous variables are presented as median (inter-quartile range). Abbreviations: PRAL: potential renal acid load, NEAP: net endogenous acid production, ER: estrogen receptor-positive, PR: progesterone receptor-positive, METS: metabolic equivalent/week, CES-D: the Center for Epidemiological Studies for Depression.

**Table 2:** Baseline characteristics according to quartiles of the PRAL score in the WHEL study (*n* = 2975).

	PRAL Score Quartiles (mEq/d)				
	Quartile 1	Quartile 2	Quartile 3	Quartile 4	*p*-Value
	<−13.7 (*n* = 771)	−13.7 to <−3.7 (*n* = 769)	−3.7 to <4.7 (*n* = 771)	≥4.7 (*n* = 770)	
NEAP (mEq/d) ^a^	27.4 (23.9–30.7)	36.4 (33.7–38.5)	43.7 (41.1–46.3)	55.4 (50.9–61.3)	<0.001
CES-D score	2 (1–3)	2 (1–4)	2 (1–4)	2 (1–4)	<0.001
Vegetable intakes (serving/d)	4.13 (2.65–5.74)	3.00 (2.05–4.00)	2.37 (1.63–3.25)	2.05 (1.40–2.83)	<0.001
Vitamin B12 (μg/d)	3.11 (1.98–4.76)	3.13 (2.12–4.55)	3.33 (2.26–4.71)	3.71 (2.58–5.20)	<0.001
General characteristics					
Age at diagnosis (years)	52.0 (47.0–58.0)	51.0 (46.0–58.0)	50.0 (45.0–57.0)	48.0 (42.0–55.0)	<0.001
Overweight and obese (%)	43.4	53.3	63.9	67.2	<0.001
Education, at or above college (%)	64.8	57.4	52.7	46.3	<0.001
Postmenopausal women (%)	84.5	80.1	80.0	73.2	0.001
Smoking status					
Past smoker (%)	44.6	43.0	44.1	43.1	0.9
Never smoker (%)	55.4	56.9	55.9	56.9	
Alcohol abstainer (%)	32.1	30.5	33.7	30.8	0.3
Physical activity (MET/week)	825 (330–1500)	630 (225–1335)	480 (150–1080)	405 (60–1080)	<0.001
Chemotherapy (%)	63.6	61.4	59.5	62.5	0.3
Radiation (%)	63.6	61.0	59.1	62.2	0.6
Hormone receptor status					
ER+/PR+ (%)	63.2	63.1	62.3	58.1	0.003
Cancer stage at diagnosis (%)					
I	38.8	36.7	38.7	38.9	0.4
II	55.4	59.6	56.7	55.0	
IIIa	5.7	3.7	4.6	6.2	
Tamoxifen use (%)	72.0	66.9	63.6	62.2	0.001

^a^ Continuous variables are presented as medians (interquartile range). Abbreviations: PRAL: potential renal acid load, NEAP: net endogenous acid production, METS: metabolic equivalent/week, ER: estrogen receptor-positive, PR: progesterone receptor-positive, CES-D: the Center for Epidemiological Studies for Depression, and WHEL: Women’s Healthy Eating and Living.

**Table 3:** Dietary acid load in relation to depression estimated using the CES-D score. CES-D scores ≥ 5 indicate depressive symptoms.

			**CES-D Score (≥5 vs. <5)**			
			**OR (95% CI)**	**OR (95% CI)**	**OR (95% CI)**	**OR (95% CI)**
Dietary acid load			Age-adjusted	Multi-model 1	Multi-model 2	Multi-model 3
**PRAL(mEq/d)**		Range				
	Quartile 1	<−19.50	Ref	Ref	Ref	Ref
	Quartile 2	−19.50 to <−6.94	1.09 (0.91–1.29)	1.01 (0.84–1.20)	1.02 (0.86–1.23)	1.01 (0.84–1.18)
	Quartile 3	−6.94 to <3.22	1.35 (1.14–1.60)	1.21 (1.02–1.45)	1.24 (1.04–1.48)	1.17 (0.96–1.43)
	Quartile 4	≥3.22	1.51 (1.27–1.80)	1.29 (1.08–1.56)	1.34 (1.11–1.62)	1.26 (1.02–1.53)
	*p* for trend		<0.0001	0.002	0.0008	0.01
**NEAP(mEq/d)**		Range				
	Quartile 1	<28.44	Ref	Ref	Ref	Ref
	Quartile 2	28.44 to <37.25	0.91 (0.78–1.07)	0.86 (0.73–1.02)	0.88 (0.74–1.04)	0.84 (0.71–1.00)
	Quartile 3	37.25 to <46.90	1.27 (1.08–1.49)	1.16 (1.98–1.37)	1.20 (1.02–1.43)	1.13 (0.94–1.35)
	Quartile 4	≥46.90	1.29 (1.09–1.53)	1.12 (0.93–1.34)	1.17 (0.98–1.41)	1.06 (0.87–1.30)
	*p* for trend		<0.0001	0.02	0.009	0.1

Covariates in the multivariable-adjusted model 1 included age at diagnosis, menopausal status at baseline, total calorie intake, physical activity, body mass index, living alone status, and number of comorbidities at baseline. Covariates in the multivariable-adjusted model 2 included covariates in model 1, plus intakes of vitamin B12. Covariates in the multivariable-adjusted model 3 included covariates in model 2, plus intakes of vegetables. PRAL and NEAL were not adjusted simultaneously. Abbreviations: PRAL: potential renal acid load, NEAP: net endogenous acid production, CES-D: the Center for Epidemiological Studies for Depression, multi-model1: multivariable-adjusted model 1, multi-model 2: multivariable-adjusted model 2, multi-model 3: multivariable-adjusted model 3, and OR odds ratio.

## Supplementary Materials

The following are available online at https://www.mdpi.com/2072-6694/12/11/3183/s1: Figure S1: Joint associations of PRAL and activity with depression.
