# Supplementary material for: Acid-Producing Diet and Depressive Symptoms among Breast Cancer Survivors: A Longitudinal Study
Source: Cancers (Basel). 2020 Oct 29;12(11):3183. doi: 10.3390/cancers12113183 (PMC7692146; doi:10.3390/cancers12113183)
Supplement: Supplementary file 1 [file cancers-12-03183-s001.pdf]

Supplementary Materials: Acid-Producing Diet and Depressive Symptoms among Breast Cancer Survivors: A Longitudinal Study

Tianying Wu, Fang-Chi Hsu and John P. Pierce

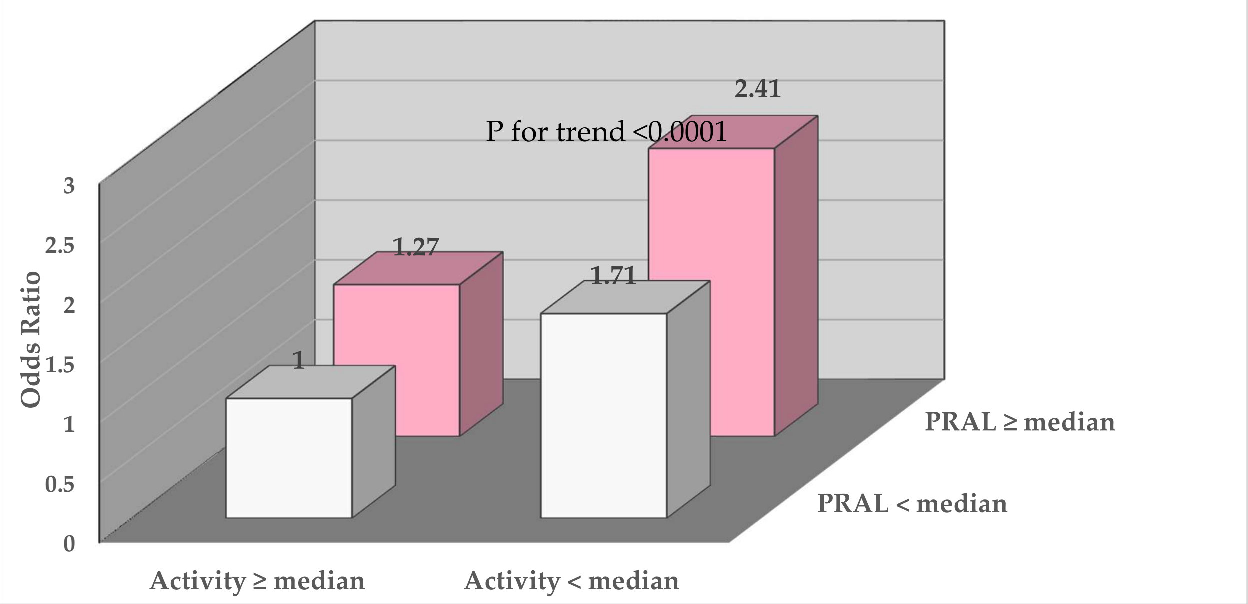

Figure S1. Joint associations of PRAL and activity with depression. Covariates in the multivariable model included body mass index, physical activity, living alone status, menopausal status at baseline, number of comorbidities at baseline, total calorie intake, and intakes of vitamin B12. Abbreviations: PRAL: potential renal acid load.

© 2020 by the authors. Licensee MDPI, Basel, Switzerland. This article is an open access article distributed under the terms and conditions of the Creative Commons Attribution (CC BY) license (http://creativecommons.org/licenses/by/4.0/).
